# Findings from Formative Research to Develop a Strength-Based HIV Prevention and Sexual Health Promotion mHealth Intervention for Transgender Women

**DOI:** 10.1089/trgh.2019.0032

**Published:** 2019-12-23

**Authors:** Christina J. Sun, Kirsten M. Anderson, Liat Mayer, Tamara Kuhn, Charles H. Klein

**Affiliations:** ^1^ Oregon Health and Science University-Portland State University School of Public Health, Portland, Oregon.; ^2^ Department of Anthropology, Portland State University, Portland, Oregon.; ^3^ dfusion, San Francisco, California.

**Keywords:** community based, HIV prevention, mHealth, qualitative, sexual health, transgender women

## Abstract

***Purpose:*** Transgender women experience significant health disparities, including increased risk of HIV infection. In this study, we examined the sexual health needs of transgender women in the context of their overall health and well-being and to identify overarching content framing strategies and content for a mobile health intervention.

***Methods:*** We conducted four focus groups and 20 individual in-depth interviews in the United States with racially and geographically diverse transgender women.

***Results:*** Four key themes were identified: structural factors as a central part of health; prioritization of transition-related care and mental health; the need for sexual health beyond preventing sexually transmitted infections and HIV; and the importance of connection and community.

***Conclusions:*** These themes can help inform the development of HIV prevention and sexual health promotion interventions for transgender women. The results suggest that the HIV and sexual health needs of transgender women should be addressed within the context of structural factors with a focus on resilience, community connection, and social support.

## Introduction

Transgender populations face considerable health disadvantages, ranging from poorer mental health to barriers to care.^1–3^ Transgender persons have the highest HIV incidence of any subgroup^4^; transgender women in particular experience significant disparities in HIV infection.^5,6^

A growing body of literature has described the links between high HIV rates and the structural contexts of transgender lives, including gender abuse, stigma, transphobia, culturally insensitive health care and health care barriers, and employment and housing discrimination.^7–13^ Other prevalent health conditions among transgender women can contribute to an increase in sexual health risk, including discrimination-based physical and verbal abuse, homicide, poor mental health, alcohol and drug use, and other unmet health needs resulting from limited health care access and negative health care encounters.^1,2,14–19^ Thus, there is a need for prevention activities that address the social and structural factors contributing to high HIV incidence rates among transgender women.^11,20–23^ In addition, the multiplicity of contextual factors influencing the sexual behavior of transgender women highlights the need to address areas of sexual health beyond HIV and other sexually transmitted infections (STIs) such as healthy relationships and communicating about sexual anatomy in a gender-affirming way. However, sexual health research focused on transgender women has nearly exclusively addressed HIV and STI prevention or sexual response following hormone treatment and gender-affirming surgery,^24^ a gap this study seeks to address.

Mobile health interventions may be particularly beneficial in addressing the HIV and sexual health needs of transgender women, as many experience social marginalization, live in areas without transgender-specific programming, and/or may have concerns with participating in face-to-face interventions.^5,11,25,26^ Previous research demonstrates transgender women use their phones for information gathering, socializing, and making sexual connections^27–29^—processes that can be leveraged for HIV prevention and sexual health promotion. Mobile apps can also deliver content tailored to individuals to address the heterogeneity of experiences and needs of transgender women and expand the reach of HIV prevention and health promotion programming.^30^

To develop a successful mobile app, research and best practices indicate that input from end users (e.g., transgender women) is vital, increasing both usability and credibility of the intervention.^31–33^ Such formative research can help to ensure that an app-based intervention is acceptable to potential users and addresses the needs and priorities of the community, increasing uptake and continued use of the app, ultimately leading to a greater ability to produce long-term behavior change.^33^ The purpose of this study was to understand the HIV prevention and sexual health needs of transgender women within the context of their overall health and well-being and identify overarching content framing strategies and content for a mobile health intervention.

## Methods

We conducted four focus groups from November 2017 to January 2018 and 20 individual in-depth interviews from March 2018 to August 2018 with transgender women as formative research to develop an HIV prevention and sexual health promotion app. Semistructured focus group and interview guides centered on overall well-being and connectedness, transgender health and sexual health, and Internet and social media use (Table 1). We conducted both focus groups and individual in-depth interviews because each methodology offers unique strengths and together produce a more complete understanding of community needs than either alone.^34^ Participants who provided services to other transgender women were also asked to consider the experiences of the transgender women with whom they worked in responding to the prompts. All focus groups and interviews were audio recorded and transcribed.

**Table 1.** Semistructured Focus Group and Individual In-Depth Interview Guides

Domain	Questions^*^
Well-being and connectedness	What's going well in your life [life of transgender women you work with/provide services to] today?^a,b^
	What are the biggest challenges you [transgender women] are facing today?^a,b^
	Can you think of any people, organizations, or other factors that have helped you [What do you think most helps transgender women] lead a healthy and satisfying life?^a,b^
	Who do you feel most connected to?^a,b^
	[How would you describe the networks of the transgender women you work with/provide services to?]
Transgender health and sexual health	What do you think are the most important health issues facing transgender women today?^a,b^
	What do you think are the most important sexual health issues facing transgender women today?^a,b^
	Why do think transgender women are still getting infected with HIV?^a,b^
	What are your thoughts on PrEP for transgender women? Do you know any transgender women who are on PrEP or have considered going on PrEP? Or have been on PrEP and since stopped? How did they make these decisions?^a,b^
	What are your experiences with HIV prevention programs and services? What have you liked/disliked? How might these programs and services best meet your [transgender women's] needs?^a,b^
	Where do you [your colleagues and/or clients] get information about (a) transgender health issues, (b) sexual health issues, and (c) health issues in general?^a,b^
Internet and social media use	How do you use your cell phone on a typical day and night?^a,b^
	Can you tell me a little more about how you use your phone to connect with other people?^a^
	What kind of apps and web sites do you like? Why? What features do you use and why? Which features do you not use and why?^a,b^
	Do you like apps and web site that involve role playing and you're being able to create your own scenarios?^b^
	What kinds of apps and web sites don't you like? Why not? Do you still use them despite not liking them? Why or why not?^a^
	How do you use your phone and apps to define yourself and your image? Do you tend to create your own content (texts, photos, video, etc.), share existing content, or both?^b^
	As we develop our Trans Women Connection sexual health app, what suggestions would you make?^a,b^

^*^ [] modification to the questions for participants who provided services to other transgender women.

^a^ Asked to focus group participants.

^b^ Asked to interview participants.

PrEP, pre-exposure prophylaxis.

### Participants

The eligibility criteria were age 18–59, self-identification as a transgender woman, and English speaking. Focus group and in-depth interview participants received $100 and $50, respectively. Community-based organizations in San Francisco, Miami, Atlanta, and Portland, Oregon (one in each city), recruited participants and cofacilitated focus groups. Interview participants were recruited through snowball sampling based on recommendations from our partner organizations and community advisory board. The community advisory board consisted of transgender women of color who work intimately with transgender communities. These multidisciplinary experts in transgender health work across diverse organizational structures, including community-based organizations and health care systems.

### Analysis plan

We used grounded theory and ethnographic methodologies to identify themes.^34,35^ Four coauthors (C.J.S., C.K., K.M.A., and L.M.) independently read and coded two focus group transcripts. The team then compiled a master code list, which two coauthors (K.M.A. and L.M.) used to code all transcripts with new codes added as needed after group discussion and consensus. We met biweekly during the coding process to ensure reliability among the coders, discussing and resolving by consensus all coding issues and iteratively refining our coding system and process. These discussions included how coders determined which codes to use, whether new codes were needed, whether redundant codes might be combined, and how coders decided on how much text to highlight for a given code or set of codes. ETR and Portland State University Institutional Review Board approved protocols.

## Results

Fifty-seven transgender women from 10 states participated (Table 2); 12 provided support or services to other transgender women. Nearly four-fifths (79%) of our sample were transgender women of color and all participants were 18 to 59 years old. We identified four main themes related to the health of transgender women.

**Table 2.** Participant Demographics

	Interview participants	Focus group participants
	n=20	n=37
	n (%)	
Race		
Black	10 (50)	14 (38)
Latina	1 (5)	12 (32)
White	6 (30)	6 (16)
Asian	1 (5)	5 (14)
Multiracial	2 (10)	0 (0)
Region		
West	4 (20)	16 (43)
Midwest	8 (40)	0 (0)
South	3 (15)	21 (57)
Northeast	5 (25)	0 (0)

### Employment and other structural factors are central to transgender women's well-being and health

Participants focused on the centrality of structural factors in supporting healthy lives. The most commonly mentioned topic across focus groups and interviews was employment. Even when asked directly about health issues, participants still highlighted work and financial stability.

Most respondents identified employment, housing, and other structural barriers as important contributors to HIV risk among transgender women.

If people have more food security, housing security, that might be the single biggest thing that will move the needle on that [reducing HIV transmission] (White, 25, West).

In particular, lack of employment was described as leading transgender women to engage in sex work.

Lack of employment opportunities… push a lot of trans people into survival sex work, something that they may not necessarily want to do, but the only way that they have to get income to pay for their next hotel night or their next meal, or transportation to get anywhere (Black, 38, South).

Participants, in turn, linked involvement in sex work to lack of social acceptance, particularly those who are visibly transgender.

I met one trans woman at a support group who's a working girl, and she's scared—she's scared all the time of catching something. And she's not very passable, you can tell that she's trans no matter how she does her makeup, and finding employment has been extremely hard for her. That's why she's making money as a working girl (White, 37, West).

Despite challenges, many women positively described employment or job opportunities in their own life or the lives of other transgender women. They noted that regular employment allows transgender women to achieve financial independence without relying on either sex work or social services. Several participants stressed that obtaining a legal name change can alleviate misrecognition, employment discrimination, and the health risks associated with involvement in illegal income generation activities. One participant explained the difficulty without a legal name change:

You're outing yourself to employers, so if the employer is discriminatory toward trans folks, you just outed yourself just by simply giving them your ID, therefore you don't have a job. You don't have a job now, so now you have to hustle (Black, 29, Midwest).

Women also described that having an ID with a name or gender marker that did not match their presentation opened them to discrimination when seeking health care services, even from organizations primarily serving the LGBTQ community. One woman described the reasons she and other women may feel uncomfortable being tested for HIV without an updated ID:

They [transgender women] come in looking like a woman but then show male names on the IDs to get your stuff. It becomes awkward and you get people who walk out of testing because they're like, “I'm embarrassed because I don't want to tell nobody I'm a guy, or I don't want to tell nobody I'm a girl,” you know? (Black, 37, Midwest)

### Transgender women prioritize mental health and transition-related health needs, but struggle to find knowledgeable and affirming providers

Many participants critiqued the quality of health care that most transgender women receive, noting that discrimination is common in medical settings. Participants described frequently encountering a lack of understanding from providers. For instance, one focus group participant told us,

It's very hard finding resources… just for basic health that will understand and accept and not point a finger the whole time while you're there (Miami Focus Group).

These concerns cut across regions and urban/rural divisions, with nearly all participants providing examples of how doctors and other medical staff did not interact respectfully and appropriately with transgender patients.

Even when providers demonstrate empathy, they may still not know how to provide transgender health services. Many participants reported that it was very difficult to find doctors who specialize in caring for transgender patients, and most general practice providers did not have the skills or knowledge to care for their transgender patients.

I haven't met any [doctors] that specialize in transgender medicine, so you're having to tell them what you need for medicine and everything (Black, 31, Midwest).

A number of participants talked about needing to provide information and education to their providers about how to care for transgender patients:

A 20-min appointment turned into an hour and a half while I explained to the doctor how to take care of me and trans patients in general (Black, 35, West).

Women also linked difficulties finding providers for sexual health:

I think one of the other biggest challenges with sexual health is, for trans women, being able to find an affirming ob-gyn… Finding that and just being able to get those normal check-ups that you need to maintain your sexual health is a challenge (White, 52, Midwest).

Participants were especially concerned about transition-related care, as exemplified by:

That's the biggest thing, finding someone who will get you the medication you need to transition (Black, 37, Midwest).

Participants expressed that they encountered problems related to both physician knowledge about and willingness to manage transition-related care:

Medical gatekeeping is a huge issue… where doctors aren't as willing to help you out and don't want to give you access to certain things. Even the best doctors here really aren't informed about our care for transition…and you just kind of have to wind up working together on something that neither of you are totally sure about (White, 20s, South).

However, participants across regions recognized that health care is beginning to be more accepting and knowledgeable for transgender patients:

We also see a lot of more [of] the medical field being interested in gaining adequate competent knowledge of treating transgender patients (Black, 38, South).

Importantly, it is not just individual providers who seem to be trying to provide better care for transgender patients:

By and large it feels like more and more organizations are interested in finding out how to provide good care, as opposed to being reticent (Black, 35, West).

One key area where some are seeing improvements is in transition-related care.

Lately it's been a little bit easier for people that are seeking hormone therapy because before 2–3 years ago, I'd talk to my doctor about it and she'd be pretty clueless… But I think now people who deal and work with the LGBT community, you know, especially trans, are becoming a little bit more educated (Multiracial, 19, Northeast).

These experiences highlight the complicated and evolving relationship between transgender women and health care providers. This complexity was especially evident for HIV-related care. Some transgender women repeatedly spoke of the ways they felt excluded, uncomfortable, and unseen by many HIV organizations.

I don't think they precisely meet the needs of transgender women because they class us with MSM [men who have sex with men]… They don't gender us correctly and if they are providing services for us, then the people who are providing the services have had no gender training as far as correct pronouns, and preferred names over legal names. All of that matters, and if you want to receive care, you want the environment to be inviting, you want the people to be non-judgmental because everybody's transition does not look the same (Black, 40s, South).

Other participants suggested that they might seek out HIV specialists or an HIV clinic when looking for transgender-affirming care.

If I were looking for an affirming provider in another city, in another state, and I saw that there was an HIV-focused provider, I'd probably get in touch with them to see if they knew anything about trans health care, if they were willing to learn, because folks who are used to doing med management for HIV are used to having to challenge things, try things, look at different things, be adventurous, and also be willing to buck the system in a lot of ways because they want to help the patient first, and they're willing to do the work to get there (Black, 35, West).

In addition to hormone therapy and gender-affirming surgery, another key component of transition-related care is mental health. A majority of participants mentioned mental health, both specifically transition related and more broadly, as one of the most important health issues facing transgender women. For many, mental health was closely linked to transition and their experience as transgender people.

I feel like for me, the trans experience was—it started as a mental thing for me, which developed into a dysphoria. Which caused a conflict with my physical [health] (Black, 31, Northeast).

Several transgender women discussed the importance of mental health care during transition and feeling as though mental health aspects of transition were neglected. Many women described how they and other transgender women suffered from depression, suicidality, social anxiety, and other mental health problems, often as a consequence of discrimination, isolation, and other struggles that they faced as transgender women.

I guess mental health, it has to be the hugest one, because I have so many problems with thoughts of suicide or things like that, and I know a lot of trans women deal with that (White, 41, West).

Given these realities, participants felt that there was a significant need for mental health care in the transgender community but identified a lack of both knowledge about mental health and access to appropriate mental health care.

So many of us deal with so many different things. And I think primarily in the African American community, it is not often used as a resource, mental health, so I really promote mental health as far as having someone to talk to about things that you feel like you can't talk to anyone else about (Black, 31, Northeast).

Some participants described how mental health stigma was a barrier that prevented women from talking about mental health or receiving care.

And it's a lot in the community that girls like us don't see because they might be looked down on or they might be ashamed, or just not even feel comfortable to reach out because of their mental illness (Asian, 38, Midwest).

Participants further described challenges finding therapists who were able and willing to see them for both gender identity and other diagnoses.

Trying to find a good therapist that will help you with your transition and someone to vent to and deal with those daily problems besides being trans is hard to find… Like, my PTSD is not because I'm trans, they're two separate things (White, 37, West).

### Sexual health for transgender women is broader than HIV and STIs

Although HIV and STIs were frequently mentioned in discussion about sexual health, participants generally prioritized other sexual health concerns, including access to comprehensive and transgender-inclusive sexual education, finding an affirming gynecologist, and the challenges of dating. Women felt that current sexual education programs were neither inclusive nor adequate to educate about safer sex practices and other important sexual health information and struggled to find information about sexual pleasure that was appropriate for mental, hormonal, and surgical changes they had been through.

Participants also mentioned that sexual health discussions could be challenging for some women because of the terminology used.

Some trans women might not want to hear about safe sex because you're going to have to refer to them having a penis if they're pre-op and then that's usually hard to deal with (White, 37, West).

However, even when discussions about safer sex and protection were occurring, sexual pleasure was rarely discussed and women did not feel supported by providers in initiating discussions about sexual pleasure. Furthermore, women did not feel as though they were receiving support in navigating what could be a very fraught relationship with their bodies as they went through puberty and medical transition.

There really isn't any kind of sexual health or sexual therapy that's going into repairing trans women's relationship with their body. It can feel like a significant betrayal over the course of your life to go through the wrong puberty. You have these organs that don't make sense to you and may not work correctly because of how you understand them and how your body is. And so, while you've spent many years distancing yourself from your genitals, and then now all of a sudden after bottom surgery, you've got whole new genitals you've got to learn how to relate to and engage with. And that can be super duper challenging for people (Black, 35, West).

On the contrary, several women reported finding useful resources to help them understand and talk about sexual pleasure and their bodies from zines (i.e., historically small-circulation, self-published booklets), a sexual health app designed for cisgender women, and from (non-transgender specific) sexual education classes hosted by a local community-based organization.

Participants also described the challenge of finding sexual and romantic partners as a key sexual health issue. Some women said that difficulties in finding partners led them to expand their possible dating pool. Others explained how these challenges led to the use of online transgender-oriented web sites and apps to find dates, which could then lead women into unsafe situations. In general, difficulty finding desired partners was described as an important factor that led some women to engage in sexual behaviors that increased risk for STIs. Transgender women might agree to engage in condomless sex or secret relationships to be accepted by a partner. Desire for partner acceptance was often rooted in the feeling that a relationship, particularly with a cisgender man, affirmed a transgender woman's identity as a woman.

I feel like a lot of trans women are looking for love and acceptance, and some of that love and acceptance can be misleading, because some girls just fall in, trying to look for love or be accepted by a man, and want that relationship and then the men out here have been in relationships with other trans women and they're not being truthful with each other's status (Asian, 38, Midwest).

In this context of structural barriers and lack of gender affirmation, nearly all participants viewed pre-exposure prophylaxis (PrEP) as an effective HIV prevention option for transgender women, especially those who do not like condoms and/or are involved in sex work. Women described feeling that PrEP gave them a level of control over their sexual health that other options such as condoms simply did not.

I think that's really important to have tools in your own hands. For being able to, to be safe rather than just being at the whim of other people to like be honest or open with you (Portland Focus Group).

However, one barrier to PrEP and improved sexual health in general is the stigma that exists around PrEP and other sexual health issues.

They're [transgender women using PrEP] not willing to tell their friends that “Hey, I'm on PrEP,” and that's kind of the best advertisement for PrEP, is knowing somebody else who's on PrEP, but then there's also a lot of judgement about PrEP, because it's like, “Well, she's on PrEP, so she's easy.” (White, 52, Midwest)

This type of judgment also extended to other sexual behaviors, which could make it more challenging to have honest, open conversations about sexual health between transgender women and health care providers.

### There are opportunities and challenges to connecting with other transgender women and the LGBTQ community

Participants described a complex and shifting landscape of relationships with other transgender women and the transgender community. Most participants expressed a desire, based on their own experiences with adversity, to help others who are in need and to promote togetherness. Most women utilized online means, along with connecting with others through in-person support groups, going to national and regional transgender conferences, and relocation to create or sustain a support network. Women also started their own organizations to network and support other transgender women, which included organization focused on transgender homelessness, prison advocacy for transgender women, and education both for health and employment.

Many women discussed how mentoring had or could positively support them.

If someone was here to speak with me, especially early on in my transition, if I had someone older than me or someone more knowledgeable in this area than me, then I would have had a more easier path when it comes to my transition. But I did not have anyone to speak to or someone to ask questions to, you know, so I did a lot of things on my own (Multiracial, 19, Northeast).

Recognizing the ways that they had been helped themselves or the ways they would have wanted help, many women expressed that it was important and meaningful to mentor other women as well.

I just try to do what I can for other girls, so that even when I'm feeling bad that always seems to uprise me, helping other girls that are unfortunate. Helping girls that are homeless, and girls that are lacking in hormones or stuff like that. Just trying to be there for them (Atlanta Focus Group).

Mentoring happened formally through organizations transgender women created, worked for, or volunteered with, as well as informally online and on the street.

Although empowered by support groups and community spaces, participants nonetheless at times felt they could not trust others. Translating online connections and interactions to face-to-face relationships with other transgender women living in the same vicinity could be challenging and meant transgender women worked to get social support and connection in a combination of ways and with varying degrees of success. Even within LGBT spaces self-described as accepting and transgender friendly, some participants described them as not really welcoming transgender women or providing culturally competent services:

I almost felt like I was being discriminated against because I was trans at the GLBT, well, gay and lesbian, organization… If you went to any of the gay resources that was geared more for gay men, like HIV testing and stuff like that, like, you transitioned to try to steal the gay men, which was kind of weird. And if you went to a lesbian organization, you were the straight guy playing dress up (White, 37, West).

Despite encountering challenges from both within and outside of the LGBTQ community, transgender women we spoke with were creating connection, opportunity, and resources for themselves and working to advocate for their needs.

## Discussion

We identified four main themes in our study: structural factors as a central part of health; prioritization of transition-related care and mental health; the need for sexual health beyond STIs and HIV; and the importance of connection and community. These findings can guide the development of HIV prevention interventions for transgender women by identifying framing strategies and content for interventions.

Our findings agree with previous research about the relationship between health issues, including HIV; structural barriers such as lack of access to employment and insurance; and sex work.^6,8,11,13,36,37^ That legal name and gender marker changes were perceived barriers to employment and health services is supported by recent research documenting this relationship.^38^ In addition, as our participants reported, access to transgender-affirming, knowledgeable, and affordable health care providers and mental health care is a significant challenge for transgender women and supported by existing research.^12,15,39–41^

Our study highlights several research gaps. One is fully understanding the mental health needs and priorities of transgender women.^42^ In addition to further highlighting the need for affirming mental health providers, participants in our study emphasize that mental health care must address concerns beyond transition. While mental health care was a priority for the women we spoke to, they perceived that the importance of mental health care was not emphasized enough by either providers or transgender women, and that stigma within the transgender community against mental illness was a barrier to accessing care.

Another gap that our study identified is the lack of attention to other sexual health concerns, including sexual pleasure and relationship with the body throughout transition. Women in our study pointed out that sexual health for transgender women has overwhelmingly focused on HIV and STIs. Participants also described sexual education as continuing to exclude affirming discussion of transgender identity and sexual health. In a recent study, only 10% of LGBTQ high school students who received sexual education reported positive transgender-related content in the curriculum.^43^ Women in our study called for inclusive sexual education for both transgender youth and adults to address this gap.

One additional gap is how social support networks are formed. Research on transgender community dynamics has been limited, tending to focus on political movements (e.g.,^44,45^) rather than the day-to-day social networks and support, which our study suggests are important for intracommunity resilience and creativity. Our participants observed a lack of social connection and feelings of isolation, which taken with previous studies is linked to reduced quality of life.^46^ Many participants described relying on online platforms for support and assistance. Understanding the formation of and challenges in joining social support networks can reveal how to support the creation of healthy networks and how these networks can promote health.

Future research could examine how transgender women create communities, how transgender women support each other's lives and sexual health, and how social support enhances their ability to live healthy lives. In addition, given the role of structural factors, researchers could elucidate how transgender women respond to structural factors and how transgender women manage the influence of structural factors, particularly on sexual health outcomes. Future intervention research could focus on identifying and testing practical strategies to alter the context of transgender women's overall health and well-being (i.e., structural interventions) and assist transgender women in coping with structural barriers.

There are several limitations to our research. Nearly all of the women who participated lived in a metropolitan area, with sizable clusters in a few cities. Transgender women in rural areas or other cities may have a different experience. Recruitment occurred through snowball sampling, so many of the women were connected to the same services or community centers. Nearly all of the women who participated had some connection to an LGBTQ- or transgender-specific program. Women without these connections may face different barriers to accessing services. Finally, most women were in their 20s and 30s, although the oldest participants were in their 50s. Older or younger transgender women may have different experiences and health priorities.

Despite these limitations, these data have implications for and can inform the development of HIV prevention and sexual health promotion interventions. These findings suggest specific content framing strategies and content. For example, participants discussed the difficulty of not feeling welcomed in LGBT spaces or finding culturally competent services (theme 4). In response to this need, in our prototype HIV prevention and sexual health promotion app, we developed an interactive resource map that displays and provides details about providers and organizations, including user ratings and reviews (for more details see Sun et al.^47^). The identification of new content is another example of how the findings guide intervention development. As part of the third theme, participants described challenges of finding sexual and romantic partners as a key sexual health issue and we have refined and added to the curriculum, which we would not have done without the insights from the formative research participants.

Our study affirms that transgender health promotion can be best addressed using a syndemic framework that recognizes structural factors impacting transgender women as well as community resilience in the face of these challenges. We found that addressing HIV and sexual health by framing them in a broader scope and within their contexts of structural factors, including individual and community resilience and intersectionality, seems to resonate more and build on transgender women's strengths. In our research, discussions overwhelmingly returned to connection, social support, and a focus on empowerment and confidence, within which a discussion of sexual health would then take place. Given that most women consider transition-related health and mental health as their highest health priorities, HIV prevention and sexual health promotion could be integrated with these types of care.

## Abbreviations Used

MSM: men who have sex with men
PrEP: pre-exposure prophylaxis
STI: sexually transmitted infection
